# The HNF4A-CHPF pathway promotes proliferation and invasion through interactions with MAD1L1 in glioma

**DOI:** 10.18632/aging.205076

**Published:** 2023-10-17

**Authors:** Haitao Luo, Kai Huang, Mengqi Cheng, Xiaoyan Long, Xingen Zhu, Miaojing Wu

**Affiliations:** 1 Department of Neurosurgery, The Second Affiliated Hospital of Nanchang University, Nanchang, Jiangxi Province, China; 2 Science Research Center, East China Institute of Digital Medical Engineering, Shangrao, Jiangxi Province, China; 3 Institute of Neuroscience, Nanchang University, Nanchang, Jiangxi Province, China; 4 Department of Health Management Medicine, The Second Affiliated Hospital of Nanchang University, Nanchang, Jiangxi Province, China

**Keywords:** CHPF, MAD1L1, HNF4A, cell cycle, glioma

## Abstract

Chondroitin polymerizing factor (CHPF) is an important glycosyltransferases that participates in the biosynthesis of chondroitin sulfate (CS). Our previous study showed that silencing CHPF expression inhibited glioma cell proliferation *in vitro*, but the molecular mechanisms by which CHPF contributes to development of glioma have not been characterized. In this study, we found that CHPF was up-regulated in glioma tissues and was positively correlated with malignant clinical pathological characteristics of patients with glioma. Silencing CHPF expression inhibited proliferation, colony formation, migration, and cell cycle of glioma cells. Moreover, silencing CHPF suppressed glioma malignance *in vivo*. Immunoprecipitation, co-immunoprecipitation, GST pulldown, and liquid chromatography-mass spectrometry (LC-MS/MS) assays were used to verify the interaction between CHPF and Mitotic arrest deficient 1-like 1 (MAD1L1). In addition, Chromatin Immunoprecipitation (ChIP)-PCR analysis showed that HNF4A bound to the CHPF promoter region, which indicated that the transcription factor hepatocyte nuclear factor 4A (HNF4A) could regulate the expression of CHPF in glioma cells.

## INTRODUCTION

Glioblastoma (GBM) is the most malignant primary brain tumor in adults, and is highly heterogeneous [1, 2]. Despite the development of surgical techniques and therapeutic strategies for treatment of glioma, the 5 years survival rate of patients with GBM is low [3, 4]. The molecular mechanisms that promote GBM malignance are unclear [5, 6]. Some studies have shown that tumor-associated genes promote tumor progression. Therefore, identification of tumor-associated genes associated with GBM, and their molecular functions, are critical to clinical diagnosis and treatment of GBM.

Chondroitin sulfate (CS) is comprised of repeating disaccharide units of N-acetyl-D-galactosamine and D-glucuronic acid residues, and is heavily sulfated [7]. Chondroitin sulfate biosynthesis is strictly regulated in cells and can significantly affect disease progression. Moreover, sulfation pattern and abundance on CS determines the biochemical properties of CS [8, 9]. Some studies that used inhibitors to decompose CS chains showed that CS played a significant role in tumor malignant progression [10–12]. Characterization of chondroitin polymerizing factor (CHPF), a unique protein factor that promotes chondroitin polymerization, might reveal the molecular functions of CS in disease [13]. Therefore, CHPF may play an important role in tumor progression.

In this study, we explored the molecular functions of CHPF and its associations with Mitotic arrest deficient 1-like 1 (MAD1L1) in glioma. The results showed that CHPF was directly interacted with MAD1L1 to promote glioma progression by regulating cell cycle. Furthermore, the expression of CHPF was regulated by the transcription factor hepatocyte nuclear factor 4A (HNF4A). Our results showed that CHPF promoted development of GBM and CHPF might be a promising therapeutic target.

## RESULTS

### CHPF was upregulated in glioma tissue and was associated with poor prognosis in patients with glioma

**Figure 1:**
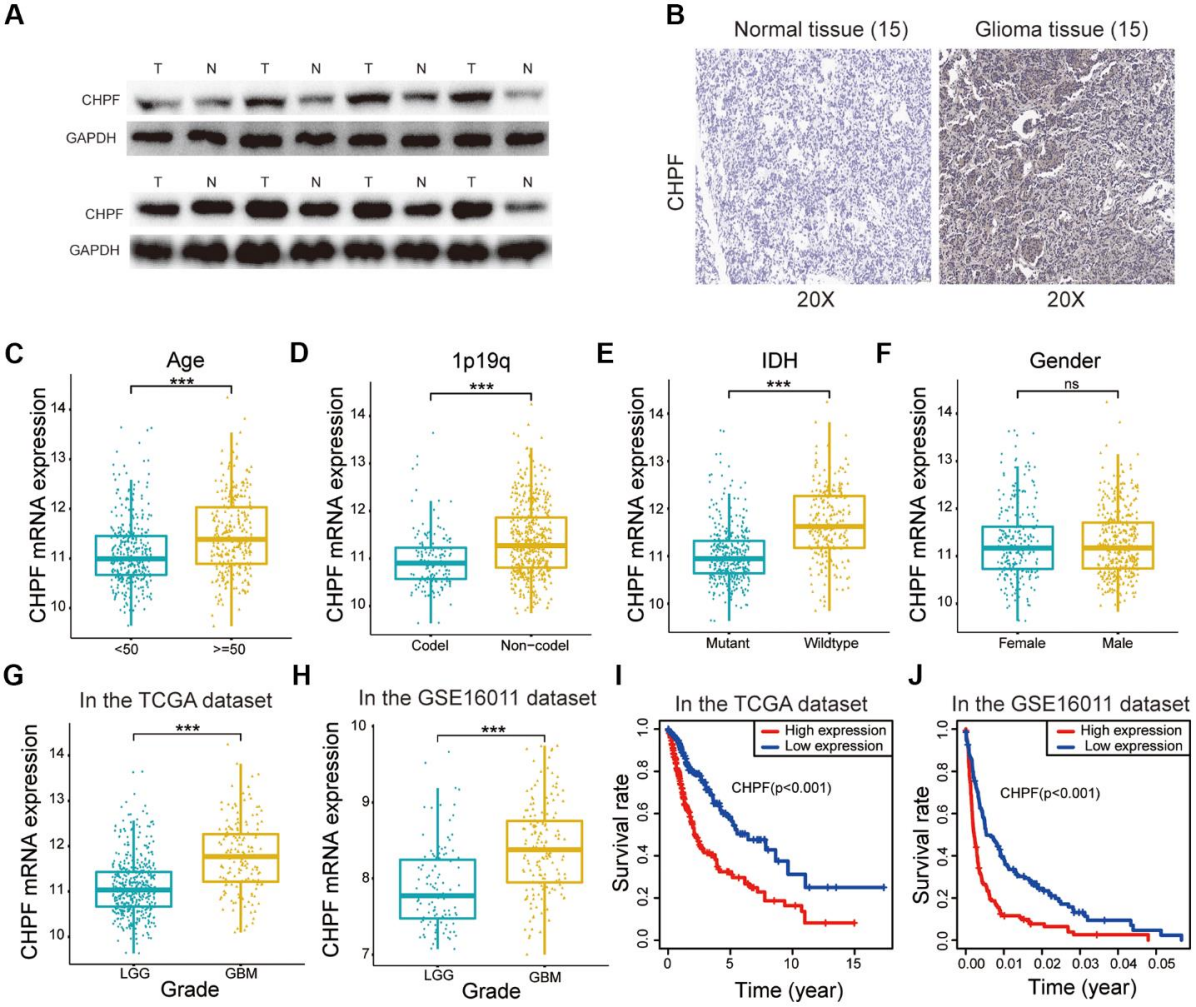
**CHPF was upregulated in glioma and served as a prognostic factor.** (**A**, **B**) The expression of CHPF in gliomas and NBTs was detected by WB and IHC assays. (**C**–**F**) Correlations between the CHPF expression and different clinical characteristics of glioma in the TCGA dataset. (**G**, **H**) Correlation between the CHPF expression and grade of patients in the TCGA and GSE16011 datasets. (**I**, **J**) Correlation between CHPF expression and the survival rate of glioma patients was analyzed by the KM curves in TCGA and GSE16011 datasets. ns P > 0.05, * P < 0.05, ** P < 0.01, *** P < 0.001.

Total protein from 15 glioma samples and 15 normal brain tissue samples was extracted to characterize the role of CHPF in glioma. Western blot analysis showed that CHPF protein expression was significantly higher in glioma samples than that in normal brain tissue (Figure 1A). Immunohistochemistry analysis also indicated that CHPF protein was expressed at higher levels in glioma samples (Figure 1B). In addition, the correlations between CHPF mRNA expression and patient survival were evaluated using data from the TCGA and GSE16011 datasets. The results showed that CHPF was expressed at significantly higher levels in GBM than in low grade glioma (LGG, Figure 1G, 1H). Furthermore, CHPF expression was positively correlated with malignant clinical pathological characteristics and was associated with a worse survival rate in patients with glioma (Figure 1C–1F, 1I, 1J).

**Figure 2:**
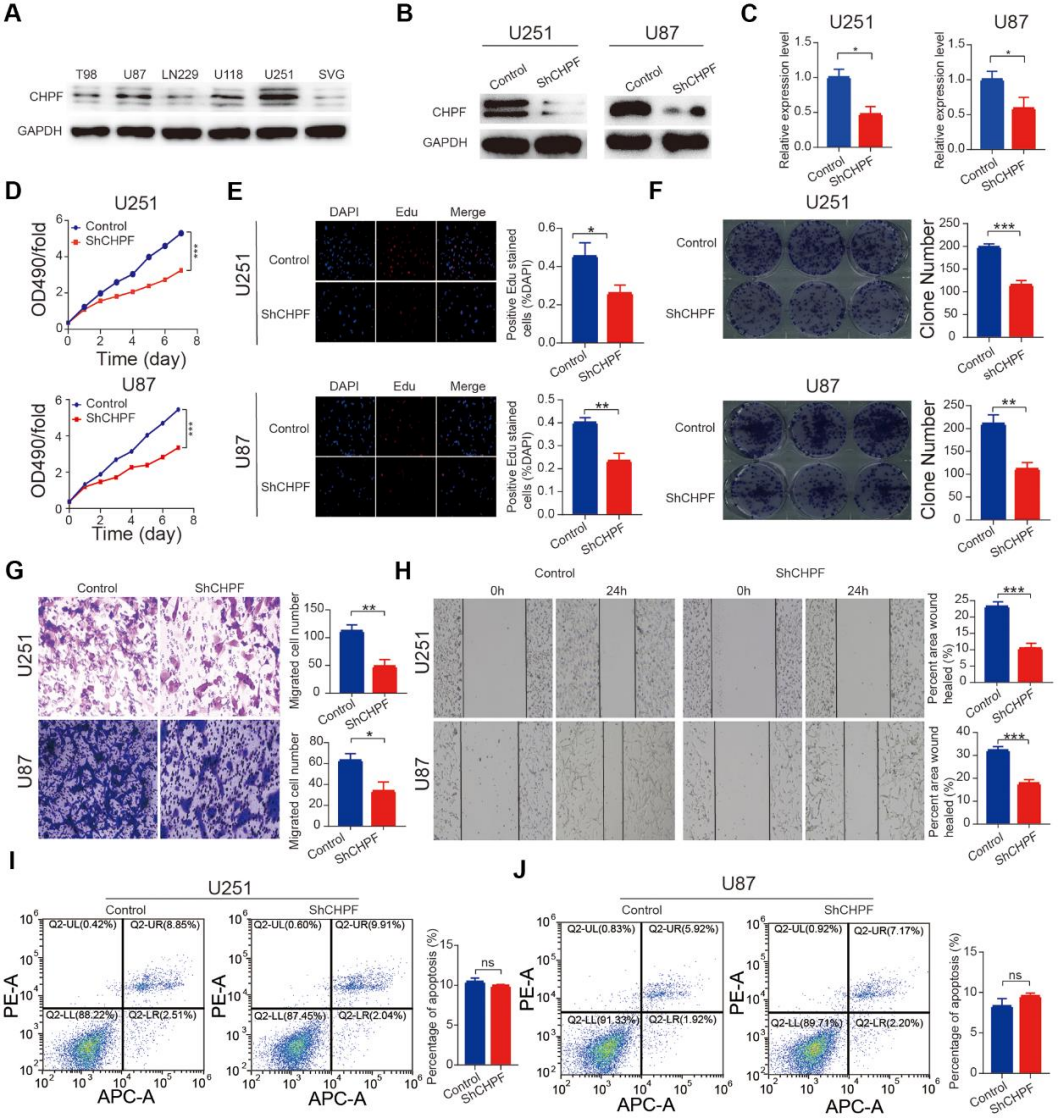
**Knock-down of CHPF inhibited glioma growth *in vitro*.** (**A**) Western blot analysis showed CHPF expression in five glioma cell lines and human normal cells. (**B**, **C**) The knockdown efficiency was verified by WB and RT-qPCR assays in U251 and U87 cells. (**D**–**F**) Effects of CHPF knockdown on cell proliferation by CCK-8, EdU and colony formation assay in U251 and U87 cells. (**G**) Effects of CHPF knockdown on invasive capacities by transwell assay in U251 and U87 cells. (**H**) Effects of CHPF knockdown on migratory ability by wound healing assay in U251 and U87 cells. (**I**, **J**) Effects of CHPF knockdown on cell apoptosis via flow cytometry. ns P > 0.05, * P < 0.05, ** P < 0.01, *** P < 0.001.

We also measured the expression of CHPF in five glioma cell lines and SVG cells. The results showed that CHPF expression was high expression in the U251 and U87 cell lines (Figure 2A).

### CHPF suppressed glioma cell proliferation, migration, and invasion *in vitro*

The U251 and U87 cell lines were selected based on the CHPF expression levels to construct a stable CHPF knock-down cell lines (Figure 2A). Knock-down of CHPF was confirmed using western blot and RT-qPCR (Figure 2B, 2C, P < 0.05). Cell proliferation was evaluated using the CCK-8 assay. The results showed that the Sh-CHPF cells grew more slowly than the control cells (Figure 2D). Results of EdU and colony formation assays showed that knocking down CHPF resulted in a significant reduction U251 and U87 cells proliferation (Figure 2E, 2F). Trans-well and wound healing experiments showed that CHPF knock-down inhibited cell migration (Figure 2G, 2H). Knocking down of CHPF did not influence apoptosis in U251 and U87 cells. (Figure 2I, 2J, P > 0.05).

### The mechanisms of CHPF induced changes in the cell cycle in glioma

**Figure 3:**
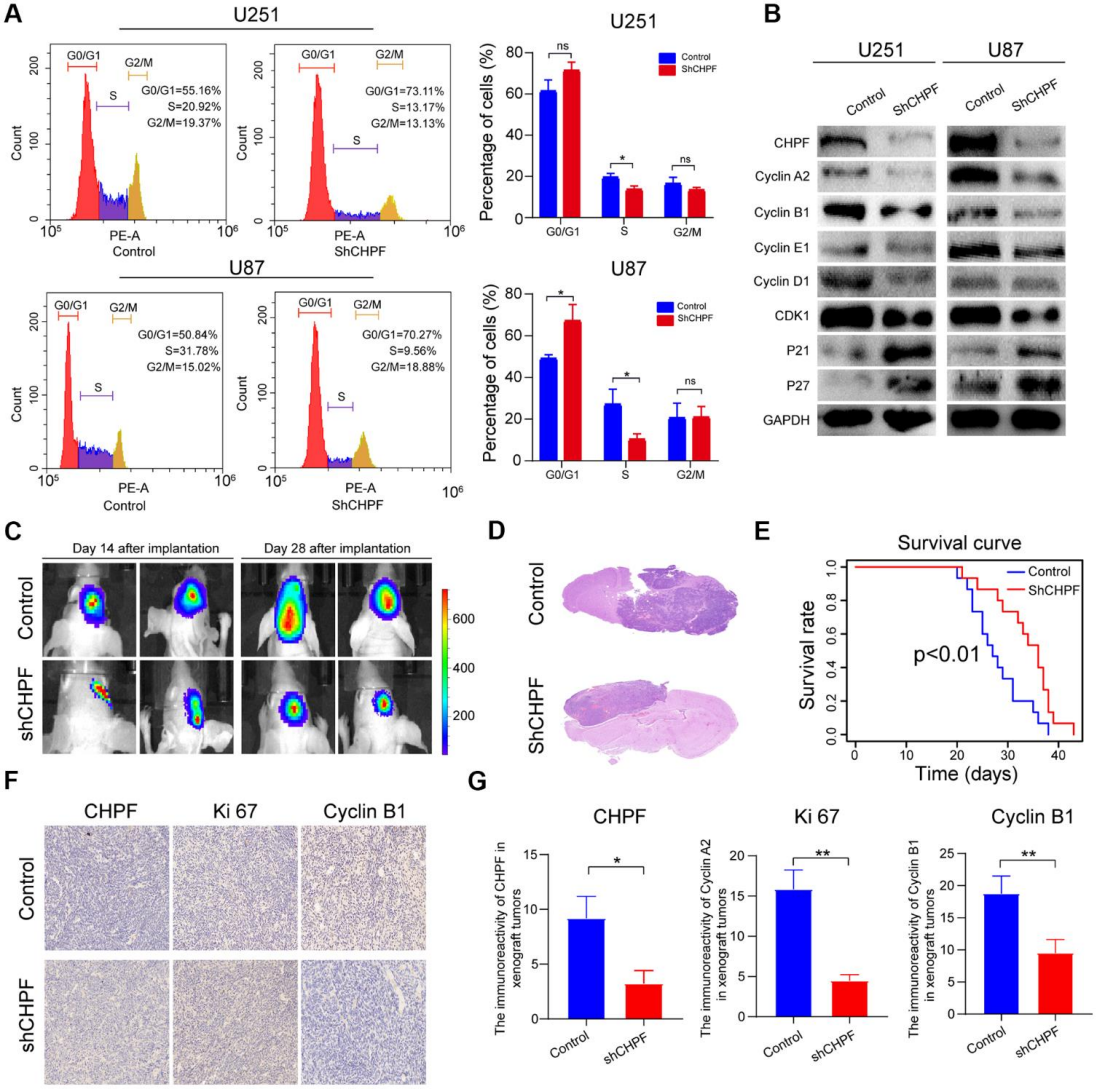
**The significant role of CHPF in cell cycle of glioma and knock-down of CHPF inhibited glioma growth *in vivo*.** (**A**) Effects of CHPF knockdown on cell cycle stages of U251 and U87 cells. (**B**) Effects of CHPF knockdown on the expression of cyclin A2, cyclin B1, cyclin E1, cyclin D1, CDK1, P21, P27 in U251 and U87 cells. (**C, D**) H&E staining of brain sections demonstrated a significant decrease in tumor volume after CHPF-knockdown at 30 days post implantation. (**E**) Kaplan-Meier survival curves showing a significant increase in median survival of CHPF-knockdown tumor-bearing mice. (**F**, **G**) The tissue micrographs of the two groups and the representative micrographs of the immunohistochemistry using antibodies against CHPF, Ki67, Cyclin B1 were shown. ns P > 0.05, * P < 0.05, ** P < 0.01, *** P < 0.001.

Previous studies showed that CHPF expression was associated with the cell cycle [14, 15], and cell proliferation is usually positively associated with cycle-related changes. Knock-down of CHPF inhibited S conversion of glioma cells (Figure 3A). Furthermore, western blot analysis showed that the expression levels of cyclin A2, cyclin B1, cyclin E1, cyclin D1, and CDK1 were reduced in the U251-sh-CHPF and U87-sh-CHPF subgroups. In contrast, the expression levels of P21 and P27, which are cell cycle inhibitors, were increased in response to CHPF knock-down in glioma cells (Figure 3B).

### Knock-down of CHPF inhibited glioma growth *in vivo*

To verify the results of the *in vitro* cell function experiments, a GBM patient–derived xenograft (PDX) model was constructed. Thirty mice were randomly sorted into U87-sh-CHPF and control subgroups. The bioluminescence images taken after, and 28 days after implantation revealed that the tumors were much smaller in the U87-sh-CHPF group, when compared to those who received control U87 cells (Figure 3C), and the H&E staining analysis also showed that the glioma tumors in the U87-sh-CHPF group grew more slowly than those in the control subgroup (Figure 3D). As shown in the survival curves, mice in the U87-sh-CHPF group had higher survival rates than those in the control subgroup (Figure 3E). Immunohistochemistry analysis showed that the protein expression levels of Ki-67 and Cyclin B1 were low in the U87-sh-CHPF subgroup (Figure 3F, 3G). There results showed that CHPF played an important role in promoting glioma tumorigenesis *in vivo*.

### CHPF interacted directly with MAD1L1

**Figure 4:**
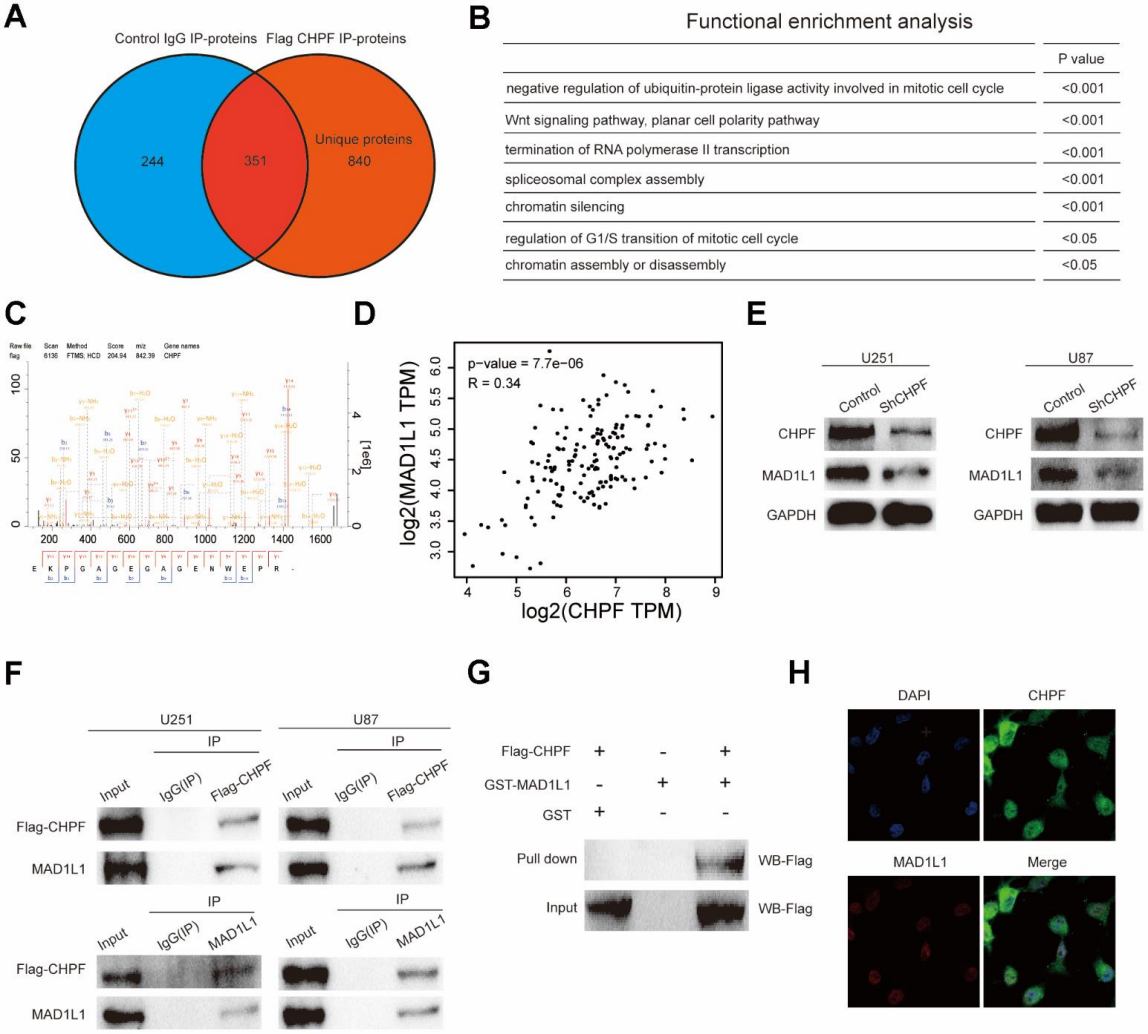
**Verify the mutual regulation relationship between CHPF and MAD1L1.** (**A**) The proteins potentially interacted with CHPF by IP and LC-MS/MS assays. (**B**) Gene ontology analysis of potentially interacted proteins with CHPF. (**C**) The result of IP and LC-MS/MS assays. (**D**) CHPF expression was positively associated with MAD1L1 expression in GBM based on GEPIA dataset. (**E**) Effects of CHPF knockdown on inhibiting MAD1L1 expression in U251 and U87 cells. (**F**) Co-IP assays were conducted to verify the interaction between CHPF and MAD1L1 in U251 and U87 cells. (**G**) The interaction between CHPF and MAD1L1 was verified using GST pull-down assay. (**H**) Immunofluorescence experiment revealed the location of CHPF and MAD1L1 in 251 cells.

To further characterize the biological functions of CHPF in the development and tumorigenesis of gliomas, we analyzed proteins in the U251 cell line that potentially interacted with CHPF using IP and LC-MS/MS assays (Figure 4A, 4C). This analysis resulted in identification of 840 unique proteins that interacted with CHPF in U251 cells. Gene ontology analysis showed that these proteins were enriched in cell cycle related pathways, such as negative regulation of ubiquitin-protein ligase activity involved in mitotic cell cycle, chromatin silencing, regulation of G1/S transition of mitotic cell cycle, and chromatin assembly or disassembly (Figure 4B). Among the potential binding proteins, we chose MAD1L1 for additional characterization based on reports that MAD1L1 was involved in regulating the spindle assembly checkpoint [16–19]. We used public datasets and western blot analysis to evaluate the associations between CHPF and MAD1L1. The results showed that CHPF expression was positively associated with MAD1L1 expression in GBM in GEPIA datasets. Furthermore, knock-down of CHPF inhibited the expression of MAD1L1 in U251 and U87 cells (Figure 4D, 4E). The interaction between CHPF and MAD1L1 was verified using co-IP, GST pull-down assay, and immunofluorescence. The results of these analyses showed that CHPF and MAD1L1 co-immunoprecipitated (Figure 4F), and direct binding between CHPF and MAD1L1 was confirmed by the GST pull-down experiment (Figure 4G). In addition, CHPF was localized in the cytoplasm and nucleus, and MAD1L1 was localized in the nucleus (Figure 4H).

### MAD1L1 played a significant role in CHPF-mediated proliferation and invasion in glioma

**Figure 5:**
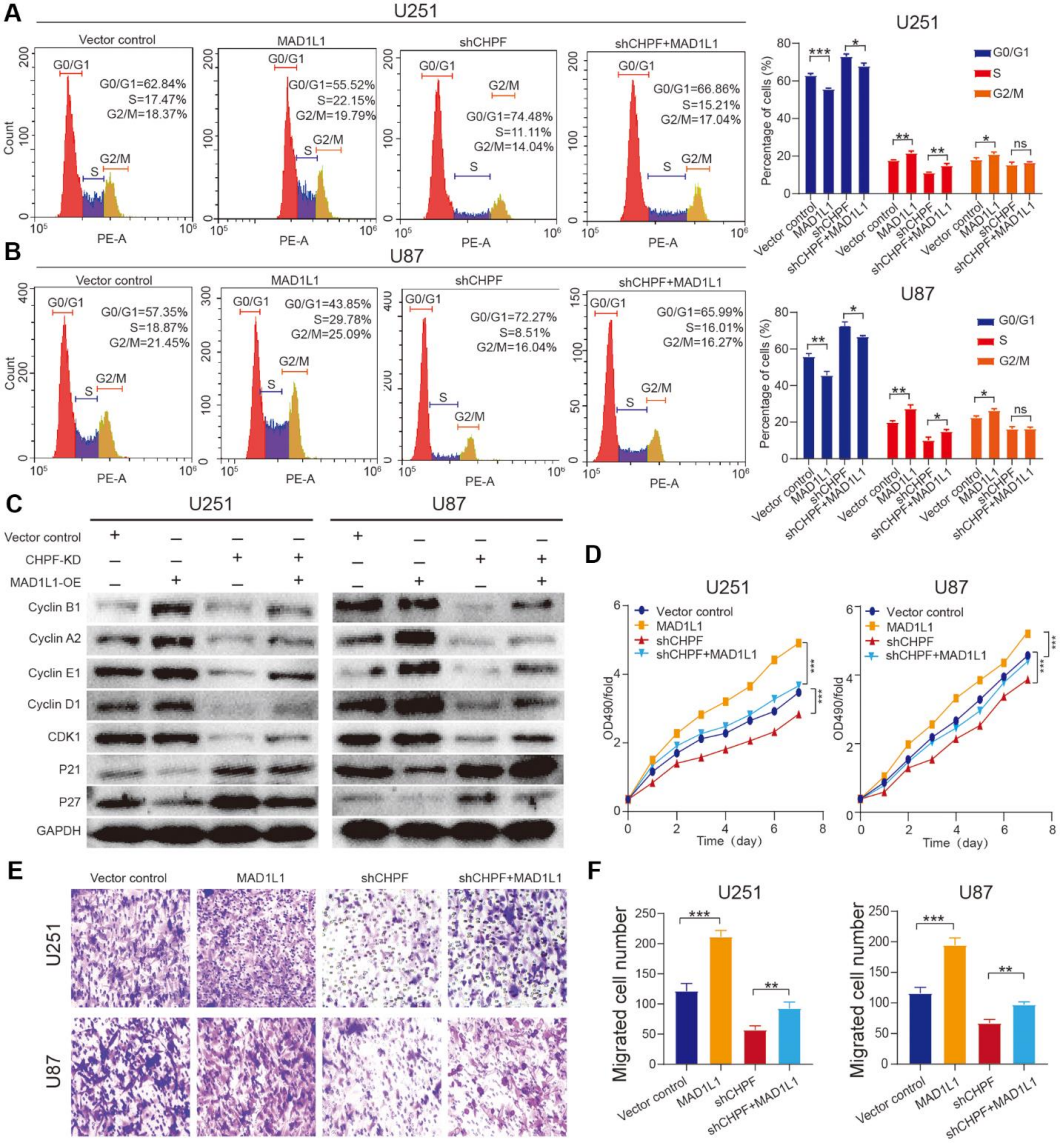
**Overexpression of MAD1L1 partially rescued the diminished proliferative and invasive ability caused by silencing CHPF expression in glioma.** (**A**, **B**) Flow cytometry assay showed the effects of MAD1L1 overexpression on U251 and U87 cells S conversion. (**C**) Western blot analysis showed the effects of MAD1L1 overexpression on the change of cycle-related proteins expression in U251 and U87 glioma cells. (**D**–**F**) CCK-8 and transwell assay showed the effects of MAD1L1 overexpression on U251 and U87 cells growth and migration. ns P > 0.05, * P < 0.05, ** P < 0.01, *** P < 0.001.

To further explore the role of MAD1L1 in CHPF-mediated promotion of glioma development and malignance, MAD1L1 was overexpressed in sh-CHPF U251 and U87 glioma cell lines. Flow cytometry results indicated that overexpression of MAD1L1 in the sh-CHPF subgroup, reversed inhibition of S conversion (Figure 5A, 5B). Western blot analysis showed that cyclin B1, cyclin A2, cyclin E1, cyclin D1, and CDK1 expression levels were relatively increased, and P21 and P27 protein expression levels were decreased in U251 and U87 cells with knock-down CHPF and overexpressed MAD1L1 compared with those in CHPF knock-down cells (Figure 5C). Furthermore, the cells in the sh-CHPF group grew more slowly than control cells, and overexpression of MAD1L1 reversed this change (Figure 5D). Transwell experiments showed that CHPF knock-down inhibited migration, and overexpression of MAD1L1 reversed this effect (Figure 5E, 5F). These results indicated that MAD1L1 was required for CHPF-mediated promotion of proliferation and invasion of glioma.

### HNF4A stimulated transcription of CHPF in glioma

**Figure 6:**
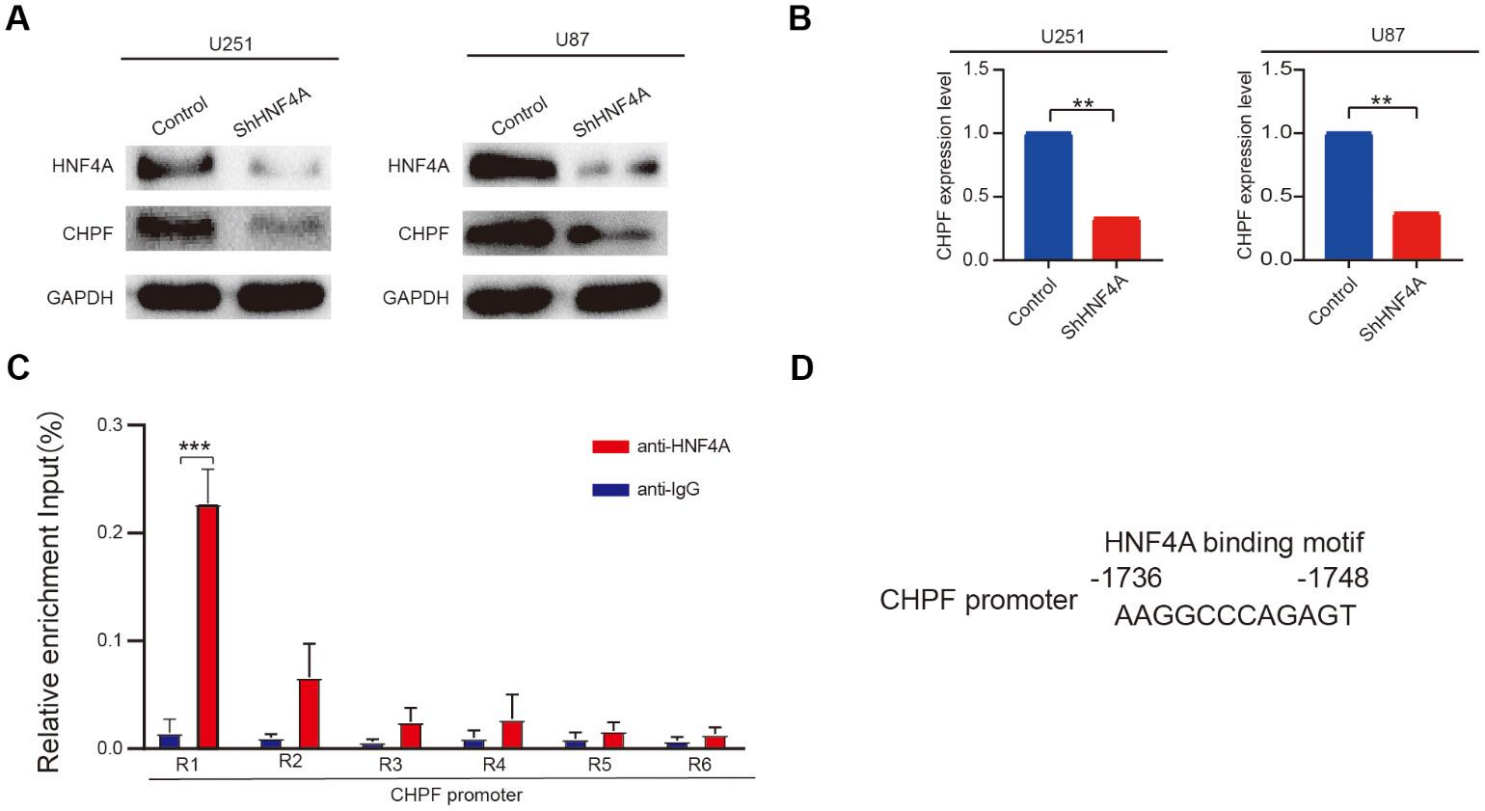
**HNF4A stimulated transcription of CHPF in glioma.** (**A**, **B**) mRNA and protein expression of CHPF in U87 and U251 cells after HNF4A knockdown. (**C**, **D**) ChIP assays were performed in U251 cells using a specific HNF4A antibody (anti-HNF4A) or a control normal IgG (anti-IgG) and detected the binding region on the promoter of CHPF was R1. ns P > 0.05, * P < 0.05, ** P < 0.01, *** P < 0.001.

Results from the USCS dataset showed that HNF4A was enriched in the promoter region of CHPF in human cells. Furthermore, HNF4A expression was positively associated with mRNA and protein expression levels of CHPF (Figure 6A, 6B). To further explore whether CHPF was a direct transcriptional target of HNF4A, we performed ChIP-qPCR analysis. The results indicated that the R1 region of the CHPF promoter (-1736 to -1748 region of the CHPF promoter) could bind with HNF4A (Figure 6C, 6D).

## DISCUSSION

Glioblastoma is the most aggressive intracranial tumor, and the OS of patients with GBM is poor, regardless of administration of standard treatment strategies [20, 21]. Therefore, there is an urgent need to characterize the molecular mechanisms of malignance of GBM. Dysregulation of gene expression influences proliferation, invasion, and apoptosis of GBM [22, 23].

Chondroitin sulfate is a polysaccharide that plays significant roles in cell adhesion, division, and neural network formation [24]. Studies have shown that at least six glycosyltransferases are involved in the biosynthesis of CS [25]. Among these, CHPF, a type II transmembrane protein, plays an important role in CS extension [26]. Some studies have evaluated the role of CHPF in initiation and progression of tumors. These studies showed that CHPF was overexpressed in lung adenocarcinoma, non-small-cell lung cancer, hepatocellular carcinoma, malignant melanoma, and breast carcinoma [14, 15, 27–34]. Our previous study showed that knock-down of CHPF inhibited glioma cell proliferation *in vitro*, but the potential molecular mechanisms of CHPF in development of glioma had not been previously characterized [14].

In this study, we found that CHPF expression was significantly upregulated in GBM and was positively associated with malignant clinical pathological characteristics of patients with glioma. Furthermore, overexpression of CHPF in glioma was confirmed using the IHC and western blot assays. The biological functions of CHPF were further explored using CHPF knock-down models in U251 and U87 cell lines. Silencing of CHPF resulted in decreased cell proliferation, migration, invasion ability and the S conversion of cell cycle. Furthermore, inhibition of glioma proliferation through silencing of CHPF was confirmed in an intracranial tumor mouse model.

Immunoprecipitation and LC-MS/MS analyses identified 840 unique proteins that interacted with CHPF in the U251 cells. The gene that encoded these proteins was heavily enriched in cell cycle-cycle related pathways. MAD1L1, a mitotic checkpoint protein [35], interacted with CHPF, and was downregulated in sh-CHPF subgroup cells. The interaction between CHPF and MAD1L1 was confirmed using co-IP and GST pull-down assays.

MAD1L1 was initially discovered during a screening analysis and was identified as an important mitotic checkpoint protein that played a role in attachment of chromosomes to the mitotic spindle by delaying separation of the replicated sister chromatids. MAD1L1 forms a heterotetrameric complex with MAD2 at unattached kinetochores, and has an evolutionarily conserved role in the mitotic checkpoint processes [36, 37]. Once the kinetochores of all sister chromatids are accurately attached to spindle microtubules, the heterotetrameric complex of MAD1L1 and MAD2 is not recruited. Decreased expression of MAD1L1 could result in aneuploidy [38]. These previous findings showed that MAD1L1 played an important role in ensuring all sister chromatids accurately attach to spindle microtubules. In our study, inhibition of glioma by downregulation of CHPF was reversed by overexpression of MAD1L1, which showed that CHPF promoted glioma malignance through regulation of MAD1L1 expression.

HNF4A is a member of the ligand-dependent nuclear receptor superfamily and is central to the regulation of intestinal development, glycolytic enzymes, lipid metabolism and epithelial mesenchymal transformation [39–41]. Studies have shown that HNF4A can bind to the promoter of specific genes to regulate their expression and can promote tumorigenesis by facilitating proliferation and invasion of many types of tumor cells, such as hepatocellular carcinoma, colorectal adenocarcinomas, esophageal cancer and neuroblastoma [42–44]. However, no studies have characterized the molecular mechanisms of HNF4A in glioma. In this study, we showed that HNF4A was enriched in the promoter region of CHPF and stimulated the expression of CHPF in glioma.

## CONCLUSIONS

In summary, the results of our study showed that CHPF was overexpressed in glioma and was positively associated with the malignant clinical pathological characteristics of patients with glioma. Furthermore, CHPF promoted glioma tumorigenesis through direct interaction with MAD1L1 and regulation of MAD1L1 expression. Finally, we showed that HNF4A stimulated transcription of CHPF. These findings indicated that CHPF may be a promising therapeutic target for GBM.

## MATERIALS AND METHODS

### Clinical tissues

Glioma and normal brain tissues (NBTs) were obtained during surgical resection at Department of Neurosurgery, the Second Affiliated Hospital of Nanchang University. The use of gliomas and NBTs was approved by the institutional ethics committee of Nanchang University (The Examination and Approval No. Review [2016] No. (122)). All patients provided written informed consents. The tissues were immediately snap-frozen at -80° C after surgical resection.

### Cell lines and cell culture

We purchased the glioma cell lines (U118, U251, U87, T98, and LN229), human normal cells (SVG) and HEK293 T cells from the Procell Life Science and Technology Co., Ltd (Wuhan, China). All cells were cultured in Dulbecco’s modified Eagle’s medium (DMEM) with 10% fetal bovine serum (FBS) and 1% penicillin at 37° C in a 5% CO2 atmosphere.

### Cell transfection

Lentivirus and plasmid were purchased from Shanghai Jikai Genentech Technology Co., Ltd and Tianjin Sheweisi Co., Ltd. Lipofectamine 2000 (Invitrogen) was used to transfect plasmids into the glioma cells. Glioma cells were seeded in 6-well plates and incubated until cells were approximately 50% confluent. Depending on the MOI value of glioma cells, appropriate lentivirus was added to each well. The cells were selected by antibiotics.

### Quantitative real-time polymerase chain reaction (qRT-PCR)

Total RNA was extracted using TRIzol Reagent (Invitrogen) and first-strand cDNA synthesis was performed using ReverTra Ace (Toyobo). We performed qRT-PCR using an ABI PRISM 7900HT Sequence Detection system as reported previously [45–47]. Glyceraldehyde 3-phosphate dehydrogenase (GAPDH) was used for normalization. The PCR primers were as follows: CHPF, Forward 5′-TCCCAAACCCAGTTTCCCT-3′ and Reverse 5′-TCCCTCCTTCCCCAACAAC-3′; HNF4A, Forward 5′ -CGTGGTGGACAAAGACAAGA-3′, Reverse 5′-ATTCTGGACGGCTTCCTTCT-3′ (downstream); GAPDH, Forward 5′ -GAACGGGAAGCTCACTGG-3′, Reverse 5′- GCCTGCTTCACCACCTTCT-3′.

### Western blot analysis

Western blot analysis was performed as reported previously. The following primary antibodies were used: anti-CHPF (ab181604; Abcam); anti-CHPF (23953-1-AP; Proteintech); anti-HNF4A (ab181604; Abcam); anti-MAD1L1 (18322-1-AP; Proteintech); anti-MAD1L1 (sc-47746, Santa Cruz biotechnology); anti-CDK1 (10762-1-AP; Proteintech); anti-Cyclin B1 (55004-1-AP; Proteintech); anti-Ki-67 (27309-1-AP; Proteintech); anti-Cyclin A2 (66391-1-lg; Proteintech); anti-P21 (10355-1-AP; Proteintech); anti-Cyclin E1 (11554-1-AP; Proteintech); anti-Cyclin D1 (26939-1-AP; Proteintech); anti-P27 (25614-1-AP; Proteintech); anti-GST (10000-0-AP; Proteintech); anti-DYKDDDDK Tag (20543-1-AP; Proteintech); anti-GAPDH (60004-1-AP; Proteintech). ImageJ software was visualized the blots and quantify bands.

### Immunohistochemistry (IHC) assay

Immunohistochemistry was performed as previously described to detect and grade CHPF, Ki-67, and cyclin B1 expression. Grades were determined by two pathologists independently. Staining intensity was graded on a scare of 0-3 (0 for negative, 1 for weak, 2 for moderate, and 3 for intense). Negative staining intensity indicated low protein expression and intense staining indicated high protein expression.

### Cell proliferation and colony formation assay

Glioma cell viability was evaluated using Cell Counting Kit-8 (CCK-8, Beyotime Biotechnology) and 5-ethynyl-2’-deoxyuridine (EdU, Beyotime Biotechnology) according to the manufacturers’ protocols. For the colony formation assay, we inoculated 800 glioma cells in the 6-well plates and incubated the cells at 37° C for 14 days in a 5% CO_2_ atmosphere. After 14 days, the cells were fixed using 4% paraformaldehyde for 30 mins, them stained using crystal violet for 1 h. The number of colonies in each well was counted using ImageJ software. Three independent experiments were performed to assess the glioma cell viability.

### Wound healing assays

Approximately 5 × 10^5^ glioma cells were plated in 6-well plates and incubated at 37° C until they formed a confluent monolayer. A plastic pipette tip was used to scratch a straight line in the monolayer. The cells were then washed with phosphate buffered saline (PBS) several times. Then, the glioma cells were incubated in serum-free medium. Photographs of migrating cells were taken at 0 and 48 h. Three independent experiments were performed.

### Transwell assay

Approximately 1×10^5^ glioma cells were plated in the upper chamber of a trans-well insert, then incubated in serum-free medium at 37° C for 2 days. After 2 days, the cells that had migrated to the lower surface of the transwell insert were fixed using 4% paraformaldehyde for 30 mins then stained with crystal violet for 1 hour. Photographs of the invaded cells were captured by the bright-field microscope. Three independent experiments were performed.

### Flow cytometry experiment

Evaluation of the cell cycle and cell apoptosis was performed as previously described. Glioma cells were stained with propidium iodide (PI) for 90 mins to evaluate the cell cycle. Glioma cells were stained for 1 h with annexin V-APC to detect apoptotic glioma cells.

### Protein mass spectrometry

Total protein from U251 cells was prepared for immunoprecipitation and subjected to liquid chromatography-mass spectrometry (LC-MS/MS) assay by Micrometer Biotech Company (Hangzhou, China). Total proteins were immunoprecipitated using anti-Flag-CHPF or anti-IgG at 4° C for 12 h. The immunoprecipitated proteins were separated using SDS-PAGE, and the protein bands were collected for LC-MS/MS analysis. The candidate proteins that interacted with CHPF are shown in Supplementary Table 1.

### Co-immunoprecipitation (Co-IP)

Immunoprecipitation lysis extraction buffer with protease inhibitors and phosphatase inhibitors were used to extract proteins from glioma cells transfected with flag-CHPF. Proteins A/G magnetic beads were incubated with anti-flag-CHPF or anti-MAD1L1 antibodies at 4° C for 90 mins, then washed three times. The antibodies were then immunoprecipitated at 4° C for 12-20 h. Then, protein A agarose and the protein-antibody complexes were incubated at 4° C for 4 h, then washed five times. The immunoprecipitation complex with protein A agarose was then isolated. Western blot was used to detect the immunoprecipitation complex.

### GST pull-down assay

The GST pull-down assay was performed according to standard procedures. MAD1L1-GST-OE and GST-OE plasmids were transduced for overexpression by IPTG in E.coli. Protein was extracted and incubated with glutathione agarose (21516, Pull-Down Kit, Thermo scientific) as the bait protein. Flag-CHPF fusion protein was extracted from HEK293T cells transfected with flag-CHPF lentivirus and used as the prey proteins. The prey protein was added to a column containing the immobilized bait protein. The column was incubated at 4° C for 2 h on a rotating platform. The column was then washed five times using PBS and elution buffer was used to collect the protein complex. Western blot was used to detect the immunoprecipitation complex.

### Immunofluorescence and confocal microscopy

Glioma cells were incubated on chamber slides at 37° C, then fixed using 4% paraformaldehyde for 30 mins. The slides were then washed three times with cold PBS. After washing, the cells were permeabilized with 0.1% Triton X-100 for 15 mins and blocked with 5% bovine serum albumin for 30 mins at room temperature. The glioma cells were then incubated with anti-CHPF and anti-MAD1L1 antibodies at 4° C for 12 h. The chamber slides were then washed with cold PBS three times. After washing, the cells were incubated with fluorescence-labeled secondary antibodies, and counterstained with 6-diamidino-2-phenylindole (DAPI). A confocal microscope was used to visualize fluorescence and capture images.

### Chromatin immunoprecipitation (ChIP) experiment

Chromatin immunoprecipitation was performed in U251 cells using a ChIP assay kit (17-295, Millipore) according to a previous study [48, 49]. The set ChIP qPCR primers were as follows: a. Forward: 5’-ACTCTTGCTGCAAAGCCACT-3’ and Reverse: 5’-GGTCACACACATCCATCCAG-3’; b. Forward: 5’-CACAGCAGCTCTGATTACCG-3’ and Reverse: 5’-TTGTGAGGATGTGTTTGGCTA-3’;c. Forward: 5’-CGACTCCAAACAAACAGCAC-3’ and Reverse: 5’-TGTCCACTGTCCTTGCTGAG-3’; d. Forward: 5’-CTCAGCAAGGACAGTGGACA-3’ and Reverse: 5’-CAGCCCAGGTAAATCTTGGA-3’; e. Forward: 5’-GAGGCAGCCAAGAGATTCAT-3’ and Reverse: 5’-CGCCGACACCATTCTCTC-3’; f. Forward: 5’-GCCTGAGGAAGGGGAAGG-3’ and Reverse: 5’-TACCGGAGAGGGAGGAGAAG-3’.

### Public data acquisition

RNA-seq transcriptome data and related clinical data were obtained from The Cancer Genome Atlas (TCGA) (https://exnabrowser.net/hub/) and GSE16011 (www.ncbi.nlm.nih.gov/geo/query/acc.cgi?acc=GSE16011) datasets. The RNA-seq data were normalized in this study.

### *In vivo* studies

Four-week-old nude mice were purchased from the Shanghai Experimental Animal Center of the Chinese Academy of Sciences and randomly divided them into two subgroups. Glioma cells were orthotopically implanted into the brains of nude mice as previous described [50–52]. Brains tissue sections were prepared and subjected to hematoxylin and eosin (H&E) and IHC staining. Tumor growth was determined by bioluminescence imaging. The company of bioluminescence imaging is PerkinElmer Inc, and the brand of instrument is IVIS Lumina XR Series III. The animal experiments were approved by the Institutional Animal Care and Use Committee of the Second Affiliated Hospital of Nanchang University.

### Statistical analysis

Unpaired analyses between two groups were performed using Student’s t-test and comparisons among three or more groups were performed using ANOVA. Survival analysis was performed using Kaplan-Meier curves. Statistical analysis was performed using SPSS 20.0 and Graph-Pad Prism v.8 software. The data are presented as the mean ± SD. P < 0.05 was considered statistically significant. Three independent experiments were performed for each experiment.

### Availability of data materials

All the data are available upon reasonable request from the corresponding authors.

## Supplementary Material

Supplementary Table 1
